# Symbiosis and the Anthropocene

**DOI:** 10.1007/s13199-021-00794-0

**Published:** 2021-09-03

**Authors:** Erik F. Y. Hom, Alexandra S. Penn

**Affiliations:** 1 Department of Biology and Center for Biodiversity and Conservation Research, University of Mississippi, University, MS 38677 USA; 2 Department of Sociology and Centre for Evaluation of Complexity Across the Nexus, University of Surrey, Guildford, Surrey, GU2 7XH UK

**Keywords:** Coevolution, Anthropogenic change, Climate change, Symbiogenesis, Invasive species, Farming, Agriculture, Fermented foods, Robustness, Homeostasis, Wicked problems, Biodiversity

## Abstract

Recent human activity has profoundly transformed Earth biomes on a scale and at rates that are unprecedented. Given the central role of symbioses in ecosystem processes, functions, and services throughout the Earth biosphere, the impacts of human-driven change on symbioses are critical to understand. Symbioses are not merely collections of organisms, but co-evolved partners that arise from the synergistic combination and action of different genetic programs. They function with varying degrees of permanence and selection as emergent units with substantial potential for combinatorial and evolutionary innovation in both structure and function. Following an articulation of operational definitions of symbiosis and related concepts and characteristics of the Anthropocene, we outline a basic typology of anthropogenic change (AC) and a conceptual framework for how AC might mechanistically impact symbioses with select case examples to highlight our perspective. We discuss surprising connections between symbiosis and the Anthropocene, suggesting ways in which new symbioses could arise due to AC, how symbioses could be agents of ecosystem change, and how symbioses, broadly defined, of humans and “farmed” organisms may have launched the Anthropocene. We conclude with reflections on the robustness of symbioses to AC and our perspective on the importance of symbioses as ecosystem keystones and the need to tackle anthropogenic challenges as wise and humble stewards embedded within the system.

## Introduction

Symbiosis is an important mechanism for generating biological novelty, shaping biodiversity, and driving major transitions on Earth (Maynard Smith 1989; Margulis and Fester 1991; Szathmáry and Maynard Smith 1995; Maynard Smith and Szathmary 1997; Szathmáry 2015). Symbiotic systems have demonstrated practical importance for ecosystem functions, biogeochemical cycles (Wang et al. 2007; Beinart 2019), and ecosystem services that include those important for agriculture (e.g., symbiotic nitrogen fixation (Bohlool et al. 1992; Peoples et al. 2009)), soil structure and water retention (e.g., biological soil crusts (Pietrasiak et al. 2013)), woodland and forest health (e.g., trees with arbuscular mycorrhizal fungi (Bonfante and Genre 2010; Willis et al. 2013)), human and animal health (e.g., gut/rumen microbiomes (McFall-Ngai et al. 2013; McFall-Ngai 2014)), and sustaining biodiversity (e.g., coral reef ecosystems (Blackall et al. 2015)). Symbioses and the services they provide have been essential for human society. In return, how has human society impacted these symbioses and the landscape of symbiosis in general?

The global COVID-19 pandemic has starkly revealed both the influences of human agency on ecosystems and the extent of potential consequences for society (Dinerstein et al. 2020; Rutz et al. 2020; Buck and Weinstein 2020; Ibn-Mohammed et al. 2021). Human impacts on the biosphere are widely accepted to be of such magnitude that we may have moved into a new geological era, the "Anthropocene" (Crutzen 2016), in which large scale drivers of environmental change stem from human activity (Ruddiman 2013; Lewis and Maslin 2015; Steffen et al. 2015a, 2020; Sarrazin and Lecomte 2016). These changes, which include climate change, pollution, habitat destruction, biodiversity loss, disruption of microbial processes, and an increase in invasive species, can have dramatic and widespread effects on evolutionary and ecological dynamics in the wild (Alberti 2015; Cheptou et al. 2017; Pelletier and Coltman 2018; Cavicchioli et al. 2019). The impact of these anthropogenic changes on symbiotic associations and on the formation of new symbioses is an open question gaining increasing attention (Six et al. 2011; Johnson et al. 2013; Mayer et al. 2014; Redman and Rodriguez 2017; Baker et al. 2018; Steidinger et al. 2019; Allgeier et al. 2020; Shu et al. 2020).

Given the degree of anthropogenic change (AC) that we have been witnessing along with its incontrovertible impact on iconic symbiotic systems such as corals (Vitousek et al. 1997; Hughes et al. 2017), it is pertinent to ask whether, how, and why symbioses (as opposed to other more general ecological relationships or organisms as individuals) might be particularly or differently affected by AC. We believe it is most useful to consider symbioses as constituting complex networks of relationships combining both biotic and abiotic components (for example, the chemical environment immediately around the symbionts; see Section 2 for details). Symbioses may be more fragile to AC via its impact on any of these critical relationships or multiple simultaneous aspects of the symbiotic system. Conversely, the existence within symbioses of active or homeostatic mechanisms to re-create and reconstitute these relationships may imbue a degree of resilience and environmental buffering against AC. At least several questions follow from this line of rationale: under what conditions does symbiotic association (as a life strategy) lead to improved resilience? How evolvable are symbioses? To what degree are symbiotic associations permanent and irreversible (Doolittle et al. 2014)? Which sorts of AC in particular could impact symbioses negatively? Is AC always destructive—i.e., are there certain human activities that could positively sustain or facilitate new symbiotic associations? Answers to these questions will likely entail many in-depth case studies and many more years of research. Our goal in this piece is to offer a perspective on how we might begin to approach these questions systematically, viewing AC and symbiosis through a common lens.

## Symbiosis in light of ecology and evolution

### An operational definition of symbiosis

Central to understanding the impact of anthropogenic change on symbioses is a clear definition of terms. **Symbiosis**, as we define it, is the ***shared genetic fate of two or more organisms via physical association***. This physical association establishes a spatiotemporal co-localization that imposes shared selective pressures for co-evolution, reduces the number of interactions with others (e.g., via endosymbiosis or by simple physical exclusion; cf. (Crowder and Cooper 1982; Stachowicz 2001)), and increases the reliability of repeated partner interactions that can bootstrap further co-evolution. We espouse two further conditions that we believe are important in defining symbiosis from an evolutionary ecology perspective; symbiotic partners: (1) **must share an environment** that may be **uniquely co-created**, and (2) do so **for a significant portion of at least one partner’s life cycle** (sufficient for common selection pressures to be experienced by all partners).

In our view, terms like “mutualism,” “commensalism,” and “parasitism” are fraught with difficulties: these terms describe a “snap-shot” in time, or “on-average” view, of **relationships that may be fluid** (Bronstein 1994; Johnson et al. 1997; van Baalen and Jansen 2001; Leung and Poulin 2008; Smith and Smith 2013; Regus et al. 2015; Zug and Hammerstein 2015; Shapiro and Turner 2018) that we believe should be viewed independent of the definition of symbiosis. These relationships are largely **perceived** with bias by the experimenter and rarely justified by **measurements of fitness**, which we believe are required for a proper characterization of these relationships. Moreover, simple net-sum cost-benefit analyses may be misleading as there may be a multiplicity of costs and benefits operating at once that mutually mask one another in an environmentally dependent manner (Smith and Smith 2013, 2015; Wagg et al. 2015). Trade-offs are a rule in biology (Csete and Doyle 2002; Tilman 2004; Shoval et al. 2012; Szekely et al. 2013; Cowan et al. 2014; Tilman et al. 2020), and complex trade-offs in a relationship may not be simply reducible to a single scalar value.

Net-sum relationship descriptors like mutualism and parasitism also fail to capture an important facet of partner interaction: the **degree of dependence**. Descriptors like “obligate” and “facultative” are often used, but these terms often have a “positive benefit” bias in their use that muddies terminology. For example, these terms are used in reference to mutualisms in roughly equal measure, but rarely is this the case for parasitisms—many parasitic stages are obligate, but parasites that might be facultative in their parasitism (causing harm opportunistically) might typically be referred to simply as pathogens (cf. Méthot and Alizon 2014). Obligate/facultative are rarely if ever used in the context of commensalism, in which there are apparently low costs of association and low partner dependence is assumed. Douglas (2010) has highlighted scenarios of symbiotic “addiction,” in which the degree of dependence by one partner is high, but the benefits received are negligible (Douglas 2010; Sullivan 2017). Symbiotic addiction as defined fundamentally requires a co-evolutionary trajectory in order to come into being. This underscores the intrinsic problem of discussing symbioses using “instantaneous terms” when a “whole trajectory” framework is needed. We believe it is most helpful and insightful to disentangle discussions of degree of dependence (e.g., low or high) from considerations of fitness costs and/or benefits, and to be mindful of eco-evolutionary timescales. Like relationships discussed above that are potentially fluid, the degree of dependence lies on a continuum and can vary over time (Nguyen and van Baalen 2020). Furthermore, partner dependence, fitness costs, and benefits, are all *environment- and context-dependent variables*, a critical idea that we will discuss below in greater depth vis-à-vis anthropogenic-driven environmental change.

Implicit in discussions about the *reversibility of symbioses* (often intertwined with the reversibility of cost-benefit relationships as discussed above) is a fundamental notion of **degree of integration**. Symbioses are unions that must integrate different *and* redundant aspects, particularly *functions*, of each individual with its partners. Given a co-evolutionary framework, the degree of partner integration is thus historically contingent and a consequence of selection acting on the **emergent symbiotic phenotype** (which may be considered to be an “interactor” in evolutionary terms (Booth 2014)). We currently lack a common framework for discussing or quantifying degrees of integration beyond the ideas of genome integration/reduction pioneered particularly by those studying endosymbionts and the evolution of organelles (Ochman and Moran 2001; Moya et al. 2008; McCutcheon and Moran 2011; Bennett and Moran 2015; Keeling and McCutcheon 2017). While genomic changes and metrics are critical descriptors of the degree of symbiotic integration, measures that describe the degree of functional integration would be welcome, especially since some functions may be redundantly encoded by multiple genes (Altenhoff et al. 2012; Das et al. 2016) and some genes may encode for multiple functions (Jeffery 2003; Piatigorsky 2007; Kalsotra and Cooper 2011; Kelemen et al. 2013; Brunet et al. 2018; Chen et al. 2021; Gallaher et al. 2021).

It is our perspective that **homeostasis**—the process of dynamic readjustment towards maintaining essential system variables that are subject to change—is a vital, emergent property of symbiotic systems that must be at the forefront of our thinking about how AC impacts symbioses. System homeostasis relates directly to our defining condition that symbiotic partners share and co-construct a common environment (see below). How partners actively niche-construct and maintain their symbiotic association determines the extent to which they are resilient and able to buffer against the degree and timescales of environmental change.

### On the evolution of symbiosis

**Fig. 1:**
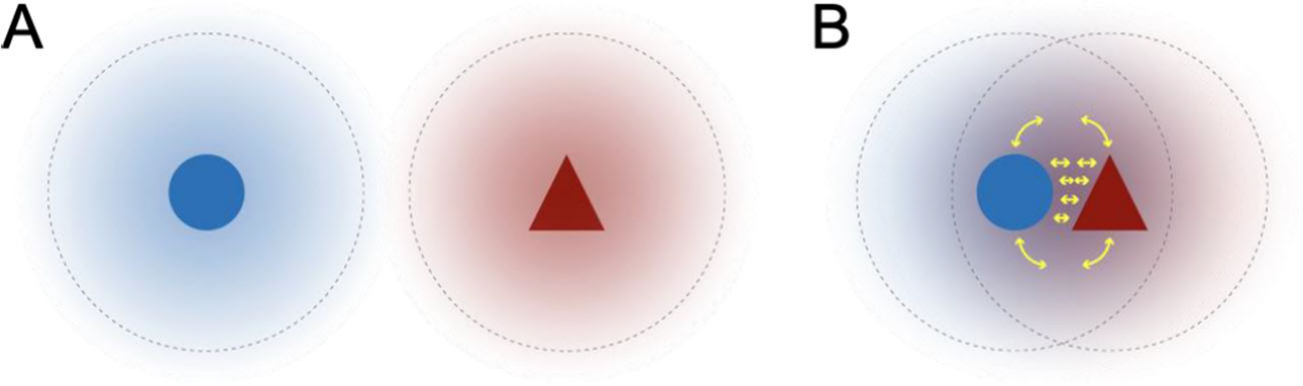
Symbiosis connects different organisms with one another through interspecific interactions to experience a common surrounding environment in space and time as well as a unique, interfacial symbiotic niche that they co-create. The perspectives of different organisms vis-à-vis the environment converge as they enter into symbiosis through physical association. (A) Each organism that is apart and separated interacts with the environment by itself, as indicated by the diffuse blue/red cloud (with dashed boundary). (B) In symbiosis, each organism comes into sustained and intimate contact so that their ‘perspective spheres’ “interfere,” creating a unique niche (purple overlap) shared by partners that supplements specific symbiotic interactions that define the association. Yellow arrows indicate interactions of each organism with each other, with the surrounding environment, or with an environment they construct together. Endosymbiosis is a specialized and more extreme union of partners, where the environment of one partner is essentially dictated by the internal environment of another and thus buffered from the external abiotic environment.

Symbiosis, and symbiogenesis (the creation of a new symbiosis) in particular, provides a unique opportunity to study processes at the nexus of ecology and evolution. Symbioses exist in a continuum linking ecological interactions with the origins of higher-level evolutionary units. This forces us to explicitly consider: interspecies interactions between unrelated organisms, aspects of self vs. non-self, interactions of each organism with the abiotic environment, and the inextricable link between biotic and abiotic system components. Our view on how abiotic and biotic factors come into play in the formation of symbioses is depicted in Fig. 1. From the perspective of a single organism, we view its “environment” to be composed of both abiotic (non-living) *and* biotic (living) components, the latter being a more dynamic (agent-driven) aspect of one’s surroundings. At the interface of symbiotic association, what one organism “sees” of the environment fundamentally changes in symbiosis through a shared environment that is co-created and maintained by partners. Asymmetric access to the external environment may be a common feature of symbiotic associations between partners of significantly different sizes, highlighting the **degree of partner dominance** as another potentially important facet of symbiosis and the possibility of “partner-driven buffering” of the abiotic environment. Endosymbiosis is the most extreme case of this whereby a partner embedded within a “host” is buffered from the external environment through internal host physiology. In the sense of partners co-creating a shared environment, symbioses embody key features of an ecosystem as a system of biotic and abiotic components defined by their mutual interactions as originally proposed by Tansley (Tansley 1935; Blew 1996; Lidicker Jr 2008). System complexity emerges from the linking and co-localization of biotic and shared abiotic niches in space and time. From the point of view of population genetics, this leads to a genotype × genotype × environment (G×G×E) interaction that can co-evolve (Thompson 2005, 2009, 2013, 2014; Morris 2018; Henry et al. 2020).

Symbioses by definition involve a close association and interaction between genetically dissimilar partners, meaning that their very existence as recognizable entities is fundamentally determined by their inter-relationships as portrayed in Fig. 1B. The nature and stability of those inter-relationships are dependent on multiple factors in both their abiotic and biotic contexts—the environment in which each partner is embedded—any of which might be disrupted by anthropogenic change. The relationships between symbiotic partners and the mechanisms evolved to reinforce them could provide a buffer against change: for example, by stabilizing a co-constructed environment or by active mechanisms to sense, find, and connect partners. From an evolutionary perspective, the degree of reliability in “reproducing” (i) a particular set of inter-relationships between partners and their environment, and (ii) the mechanisms in place to recapitulate those inter-relationships, is important for determining the extent to which a specific symbiosis constitutes a higher-level evolutionary unit of selection. This prompts us to distinguish a symbiosis from a more general or transient ecological interaction. Although out of the scope of this particular contribution, we refer the reader to several rich discussions about how symbioses or holobionts (i.e., suite of symbiotic partners (Margulis 1991)) might become evolutionary units of selection and be considered as biological individuals at a new, higher level (Margulis 1992; Queller 1997; Maynard Smith and Szathmary 1997; Booth 2014; Gilbert and Tauber 2016; Roughgarden et al. 2018; Rosenberg and Zilber-Rosenberg 2018). Nonetheless, we feel it is critical to emphasize the importance of the temporal dimension in symbiotic interactions. In the “dance” of symbiosis, the rhythms and timescales of partner processes and life cycles must be complementary and sufficiently synchronized for the association to develop and persist. Evolutionary forces will act to shape these rhythms and dynamics of symbiotic association, and how they are shaped may impact how they respond to AC. Our aim in this commentary is to explore the evidence for how symbioses in particular (as opposed to ecological interactions in general) might be affected by AC and whether general principles or broader inferences can be deduced.

## The nature of anthropogenic change (AC)

### Characterizing the Anthropocene

In the periods of time since the origins of agriculture and the beginning of the Industrial Revolution, multiple, large-scale, rapid, and accelerating changes have been observed in the Earth system (Steffen et al. 2007, 2015b, a; McNeill and Engelke 2016). Although the concept of the Anthropocene is still being formalized, the evidence for large and unprecedented human impacts on climate, biogeochemical cycles, biodiversity, habitat and land use change is widely accepted (Rockström et al. 2009; Ellis et al. 2010; IPCC AR5 Synthesis Report 2014; Steffen et al. 2015b), and has framed broad calls for new approaches to economic and societal development (see e.g., UNDP Human Development Report 2020; HM Treasury (UK) 2021). The need to respond to large scale AC is now mainstream and has started to drive policy actions to curb global megatrends that alter human-biosphere interactions over the coming century (Ribeiro et al. 2012).

Rapidly accelerating changes in both environmental and socio-economic variables are a defining feature of the Anthropocene, and one such set of variables that describe this so-called “Great Acceleration” (Steffen et al. 2015a) is presented in Table 1. These variables were chosen for their ability to represent the nature of human demands and influence on the Earth system and consequent changes in the structure and function of biogeochemical cycles and biomes.

**Table 1:** Accelerating variables that define the Anthropocene (after Steffen et al. 2015a).

Socioeconomic Trends	Earth System Trends
Population	Carbon Dioxide
Real GDP	Nitrous Oxide
Foreign Direct Investment	Methane
Urban Population	Stratospheric Ozone
Primary Energy Use	Surface Temperature
Fertiliser Consumption	Ocean Acidification
Large Dams	Marine Fish Capture
Water Use	Shrimp Aquaculture
Paper Production	Nitrogen to Coastal Zone
Transportation	Tropical Forest Loss
Telecommunications	Domesticated Land
International Tourism	Terrestrial Biosphere Degradation

In addition to these specific variables gauging AC, there has been increasing modification of ecosystems to meet human needs (Ellis et al. 2010; Williams et al. 2015), homogenization of ecological communities (Rosenzweig 2001; Didham et al. 2005), large scale biodiversity loss (Barnosky et al. 2011; Pimm et al. 2014), habitat fragmentation and destruction (Laurance 2010; Betts et al. 2019), and large scale land use change as more of the Earth’s surface and primary productivity is brought into human use (Millennium Ecosystem Assessment 2005; Smil 2011). Biodiversity changes have been accompanied by dramatic abiotic changes, including increased fossil fuel use, the synthesis and widespread environmental disposal of new chemical substances, and the reshaping of biogeochemical cycles (Canfield et al. 2010; Steffen et al. 2015a).

While environmental change has occurred throughout the history of life on Earth, many argue that AC merits special consideration as being both qualitatively and quantitatively different from prior change. In particular, anthropogenically-driven changes are: (i) rapid and potentially accelerating (Steffen et al. 2015a), so that the possibility of evolutionary escape is diminished (Ellis 2019); (ii) simultaneous in occurrence; (iii) large in magnitude relative to pre-existing earth system dynamics (Canfield et al. 2010; Steffen et al. 2015a); (iv) large in spatial scale, so that possibility of adaptation via migration is diminished (Zalasiewicz et al. 2008; Barnosky et al. 2012); and (v) often abrupt and discontinuous due to cascading failure of interconnected systems (Lenton et al. 2008). AC leads to dramatic changes in selective pressures and the availability of potential partners, so symbioses may be profoundly affected. The context-dependency of symbiotic interactions has been well known for years (Daskin and Alford 2012; Hoeksema and Bruna 2015), but a framework for understanding *mechanistically* how AC may influence this has not been well-formulated.

### A preliminary typology of AC

In order to more systematically consider the potential impacts of AC on symbioses, we have synthesized the aspects discussed above into a **typology of AC**. Given the connectedness and complexity of ecological communities and their environmental interactions, the specific “Anthropocene variables” of Table 1 do not map precisely onto a single specific 'type of change' shown below. There are numerous one-to-many and many-to-one connections, and the concepts below are inherently interdependent. However, we believe that such a typology, alongside a framework for understanding potential impacts on symbioses (Section 4.1), provides a path forward toward disentangling how AC can affect the various evolutionary and ecological aspects of symbioses that are in general distinct from other ecological relationships.

We propose the following **ten types of change** that can arise from human activity:

1. **Change in existing niches.**

Either the expansion/contraction of existing niches or the creation/destruction of niches can occur (Evers et al. 2018). For example, urbanization has led to increased availability of concrete surfaces and the built environment, and the expansion of developed residential communities has yielded a rise in artificial ponds and lakes at the expense of natural habitats.

2. **Change in homogeneity or heterogeneity of the environment.**

This may be a change in habitat or environmental diversity in space and/or in time. Increased homogeneity could arise due to the creation of new large-scale environments, e.g., as seen associated with industrial agriculture. Conversely, increased heterogeneity may arise due to disturbance-driven fragmentation of habitats.

3. **Change in ‘large-scale’ abiotic environmental variables, specifically** ***average*** **values**.

A change in the mean value or stable state of global (or higher-level) environmental variables. For example, global temperature, ocean acidification, or widespread microplastic pollution.

4. **Change in ‘smaller-scale’ abiotic environmental variables, specifically** ***average*** **values.**

This is a more focused change at a local/regional scale that may come about, e.g., through damming, deforestation, or fertilizer use.

5. **Change in the** ***variability*** **of global or local abiotic environmental variables**.

A change in the degree of variation, e.g., wider or more erratic temperature fluctuations, or more extreme precipitation patterns.

6. **Introduction of new, human-synthesized products into the environment.**

This includes new substances and their degree of release into the environment. For example, plastics, antibiotics, organophosphates, herbicides, noise, light, and increasingly, genetically engineered/synthetic organisms (Rhind 2009; Halfwerk and Slabbekoorn 2015; Schmidt and de Lorenzo 2016; Bernhardt et al. 2017; McMahon et al. 2017; Mitchell and Bartsch 2019; Saxena et al. 2020; Häder et al. 2020; Levy et al. 2020). The recent magnitude and frequency of harmful algal blooms due to increased fertilizer run-off into aquatic habitats (Kudela et al. 2015; Griffith and Gobler 2020) is a vivid reminder of this type of AC.

7. **Change in community composition.**

A biodiversity shift in compositional make-up in space or extent (range) and/or in time (Pecl et al. 2017). This may arise from the arrival or disappearance of species, the construction/management/preservation of new habitats or assemblages (e.g., urban area or farm), or increased anthropogenic mixing of species via active transportation or habitat incursion. This fundamentally alters the distributions of species and consequently their interactions.

8. **Change in interlinkages between system components.**

This captures interlinkages that may form (or disappear) at levels higher than the interaction of individual species. This may occur through decoupling of habitats (e.g., damming or dividing habitats by road/structure construction) or via coupling the previously unconnected (e.g., linking underwater oil reserves to surface waters and beaches in the Deepwater Horizon oil spill (Beyer et al. 2016), air travel linkages that connect pathogen hotspots globally (Tatem et al. 2006; Bell et al. 2021), or maritime transport that facilitates long-range dispersals (Wilson et al. 2009; Blakeslee et al. 2010; Lymperopoulou and Dobbs 2017)).

9. **Change in community “momentum” or trajectory**.

Disruption of successional processes can dramatically alter an ecosystem’s momentum or trajectory of development. For example, repeated ploughing in conventional agriculture, clearing in slash-and-burn agriculture, and the monoculture planting of climax perennial tree species in forestry (Thomas and Kevan 1993; Altieri 2002) all interfere with the “flow” of succession and actively alter an ecosystem’s trajectory.

10. **Change in selective processes**.

This may occur via “inadvertent” selection from byproducts of AC. For example, coastal urbanization may de facto lead to high light levels that negatively select against coral symbioses sensitive to light pollution (Levy et al. 2020). This may also occur via active and deliberate manipulation of selective processes such as domestication and artificial selection (Ellis et al. 2010; Williams et al. 2015). Prominent in the agricultural realm (in which many symbioses are at play), the results of large-scale artificial selection may profoundly impact symbiotic associations (Porter and Sachs 2020). In agricultural fertilization, artificial selection may occur in conjunction with herbicidal or nutrient supplementation (see #6 above) to select for specific and sometimes completely new or engineered cultivars (e.g., Bt cotton or glyphosate/Roundup-ready crops (Raman 2017)).

## How anthropogenic change impinges on extant symbioses

### A framework for how AC impacts extant symbioses

**Fig. 2:**
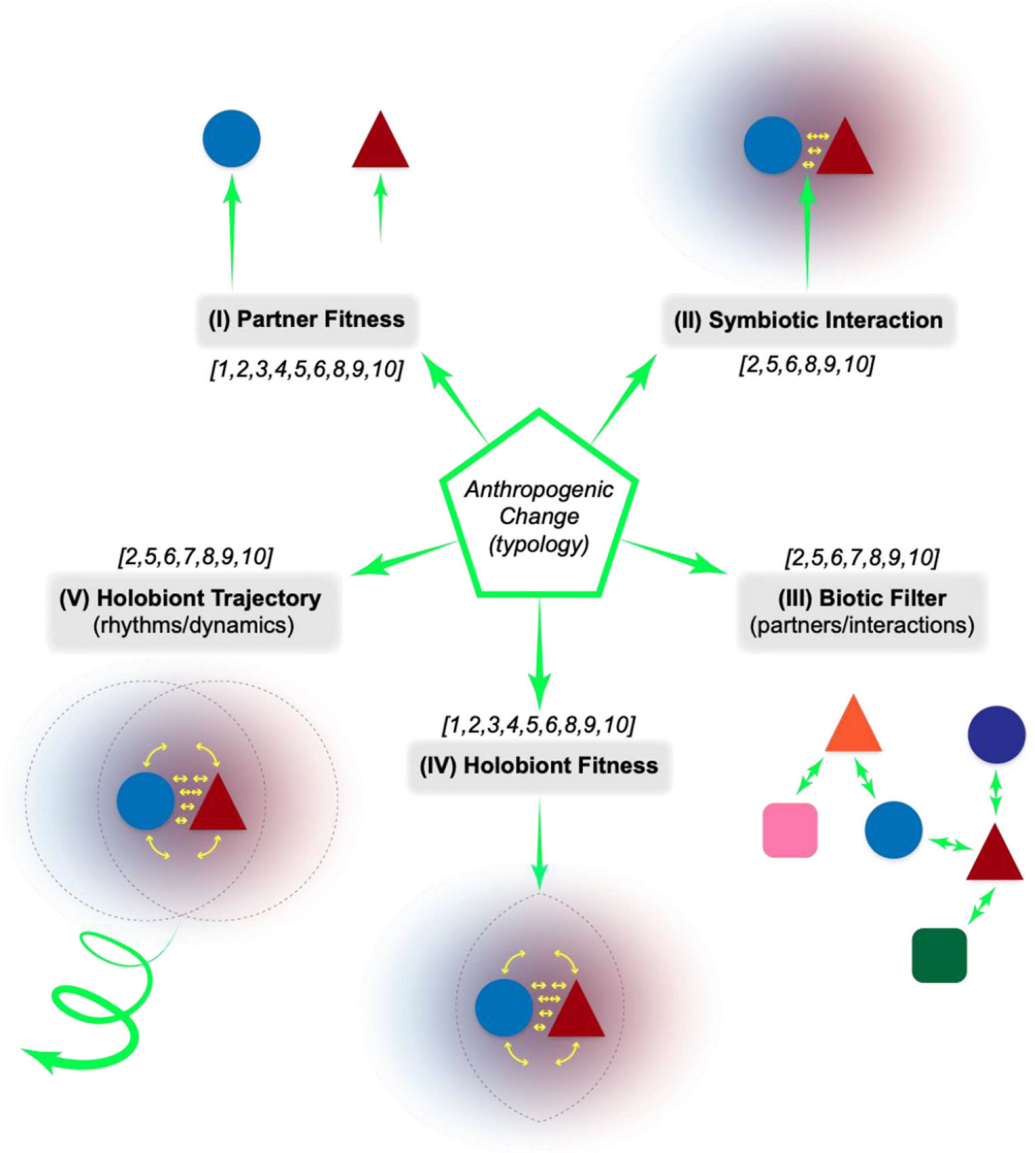
A framework for how AC impacts symbiosis. See main text for description of the 5 targets or modes of alteration. The numbers in square brackets indicate the types of AC (as described in Section 3.2) that may most commonly be mapped onto these 5 targets/modes of impact. The different length arrows in (I) highlight that differential impact on partner fitness is possible. While targets (I) through (IV) are essentially instances of the state of a symbiosis in time, target (V) is focused on an intrinsically time-dependent developmental *process*. The impact of AC on these targets are never truly independent from one another, but the utility of this framework is in delineating the *primary, most proximal* points of influence.

Given our perspective presented in Section 2.2 and in Fig. 1, we conceive of 5 major ways in which the types of AC could impact extant symbioses (Fig. 2). Importantly, we have framed this with respect to the external forces of AC from the point of view of the individual partners involved in the symbiosis (I-III) or the holobiont (IV-V):

(I) **AC impacts one or more symbionts directly, specifically altering fitness (i.e., AC alters individual PARTNER FITNESS)**

The fitness of partners could be differentially affected (Fig. 1A) and if significantly decreased, for example, could lead to the collapse of the whole symbiosis.

(II) **AC impacts a specific interaction or mode of interaction of symbiotic partners directly (i.e., AC alters the SYMBIOTIC INTERACTION)**

Changing the pay-offs of interaction (i.e., costs vs benefits) and/or the consistency of those pay-offs may fundamentally alter a partner relationship (cf. Section 2.1). AC may directly target or interfere with specific modes of partner interaction.

(III) **AC impacts the availability of partners/competitors for partner interaction and association (i.e., AC alters the BIOTIC FILTER)**

AC may change the ecological landscape of available partners (who interacts with whom) through simple structural changes in community or population composition.

(IV) **AC impacts the fitness or ecological interactions of the symbiotic entity as a whole (i.e., AC alters HOLOBIONT FITNESS]**

This can occur by changing either: (i) the niches available to the holobiont, or (ii) the degree or nature of *interaction of the holobiont with other biota*.

(V) **AC impacts the trajectory, rhythms, or dynamics of symbiosis (i.e., AC alters the HOLOBIONT TRAJECTORY)**

This mode focuses explicitly on the temporal dimension. The developmental and maintenance processes of symbioses are vital for their existence as a recognizable entity, but are distributed between partners, and hence potentially more variable and vulnerable to change. AC may interrupt the dynamics/rhythms of partnership formation or impact holobiont homeostasis, and there may be priority effects depending on *when* change occurs: disruptions of interactions early in a cascade of feedbacks may have a more profound downstream effect.

### Framework applied: case examples of how AC impacts extant symbioses

Given the framework above, we can more systematically delineate specific ways that AC can affect symbiotic relationships. Below, we provide select, illustrative examples for each mode of impact. Our intent is by no means to be exhaustive, but simply to outline how such a framework could be useful to resolve the links between cause and effect of AC on symbioses and underlying mechanisms. There are likely many other excellent examples that could have been chosen (cf. Secord 2002; Six et al. 2011; Bénard et al. 2020; Apprill 2020) and our hope is that others will be prompted to explore how this framework could be applied to specific systems of interest. As noted in Fig. 1, we consider a general arrangement whereby both symbiotic partners may have direct access to the external environment. Endosymbiosis is a more specialized (if not more extreme) case of partner coupling in which there may be greater integration and partner dependency, as well as a shielding of the endosymbiotic partner from the external environment (cf. Doolittle et al. 2014). Thus, the arrangement of endosymbiosis can limit certain modes of AC impact that we describe below. In general, modes (I)-(III) focus on a partners-as-individuals perspective while modes (IV)-(V) focus on a holobiont-as-unit perspective.

(I) **AC impacts one or more symbiotic partners directly, specifically altering fitness (PARTNER FITNESS)**

This mode of impact is common to all ecological interactions and might be the most obvious if not most common way by which symbioses can be impacted: AC directly affects the individual fitness of one or more partners. In the case of endosymbiosis, considerations of the external environment on partner fitness often simply reduce to partners that are in direct contact with and immediately affected by the external environmental variable. In the examples below, the symbioses are sufficiently “permeable” to the perturbing AC such that endosymbiont fitness is negatively impacted.

- *Air pollution and photobiont sensitivity in lichens*.

One of the most well-known early examples of anthropogenic impacts on symbioses is found with lichens. So-called “canaries-in-the-coal-mine” indicator species of regional air pollution (Richardson 1991; Seaward 1992; Conti and Cecchetti 2001; Grube 2010), lichens can be particularly susceptible to air pollutants like sulfur dioxide. Many lichen populations in urban areas worldwide have declined dramatically during periods of high and unregulated air pollution (Seaward 2004), although lichen recolonization is possible following improved air quality (Showman 1997; Ranta 2001; Dorey et al. 2019). This fragility appears to stem from a particular sensitivity of the photobiont to air pollutants (Richardson 1988; Nash III and Gries 2002; Grube 2010).

- *Thermal stress and photobiont sensitivity in corals*.

Although coral bleaching can involve a complex cascade of events and multifactorial triggers (Weis 2008; Lesser 2011; Davy et al. 2012; Hoegh-Guldberg et al. 2017; Oakley and Davy 2018), coral photobionts (symbiotic dinoflagellates) can be especially and differentially vulnerable to thermal stress (Weis 2010; Howells et al. 2012; Tolleter et al. 2013). Nevertheless, it remains unclear whether and how the coral host or photobionts could be the 'weak link' vis-à-vis many other ecosystem variables that may underlie the failure of the coral symbiosis in response to thermal stress (Hoadley et al. 2019; Suggett and Smith 2020; Drury 2020; Howe-Kerr et al. 2020; McClanahan et al. 2020).

(II) **AC impacts a specific interaction or mode of interaction of symbiotic partners directly (SYMBIOTIC INTERACTION)**

AC may directly interfere with the very nature of a symbiotic interaction in modifying the pay-off structure of association. Practically, this may be difficult to determine since this requires a careful accounting of costs and benefits to fitness and potentially a mechanistic understanding of how a symbiosis works. For example, if a symbiosis is fundamentally determined by the exchange or recognition of a specific compound X, and AC leads to a change in the environmental levels of X, this may directly interfere with the “currency” of interaction (cf. Wein et al. 2019) or with symbiotic signaling.

- *Nitrogen fertilizers, pollutants, and the disruption of the rhizobial-legume symbiosis*.

Legumes derive biologically accessible nitrogen from symbiosis with N_2_-fixing rhizobia, particularly in nitrogen poor soils. With increased agricultural deposition of nitrogen fertilizers that “inflate” the availability of soil nitrogen (Vitousek et al. 1997), the benefits of rhizobial nodulation of legumes can be greatly diminished with a concomitant deterioration of this symbiosis (Regus et al. 2017; Porter and Sachs 2020). In this case, anthropogenic nitrogen-supplementation selects against the rhizobial-legume association, shifting a symbiotic dependence of crop plants to humans to a point to which some can no longer form symbioses with rhizobia (Porter and Sachs 2020). Organochlorine pesticides, polycyclic aromatic hydrocarbons, polychlorinated biphenyls, and other chemical pollutants have also been shown to disrupt phytohormone-based signaling and recruitment of rhizobia by leguminous host plants (Fox 2004, 2005; Fox et al. 2007) (cf. mode (V) below). It would not be surprising if other symbioses in aquatic habitats are also negatively affected by exposure to such chemical contaminants.

(III) **AC impacts the availability of partners/competitors for partner interaction and association (BIOTIC FILTER)**

Another dominant mode by which AC may impact symbioses (and indeed all ecological interactions) from the point of view of individual partners is by altering the larger biotic context. This is a change in community composition or the array of organisms with which a partner could potentially interact.

- *Elevated CO*_2_ *leads to shifts in plant-microbe community composition*.

Plant-associated microbiomes play a critical role in the response of plants to AC and AC can modify plant-microbe associations. Elevated CO_2_ levels largely correlate with a greater abundance of arbuscular and ectomycorrhizal fungi in soil (Compant et al. 2010) and shifts in rhizosphere microbial communities that can alter the landscape of plant competition and community structure (Drigo et al. 2010; Jo et al. 2019). Elevated CO_2_ can cause partner plants to release more, if not different plant exudate compounds that may increase the competition among rhizosphere microbes and modify the recruitment of specific nitrogen-fixing rhizobial partners (Haase et al. 2007; Sanz-Saez et al. 2019; Prescott et al. 2020).

- *Elevated temperature alters available partners in an insect-fungus symbiosis*.

The spectrum of symbiotic fungal partners available to several species of insect hosts can be altered by rising temperatures. In beetle-fungus symbioses, higher temperatures select for warm-acclimated fungal partners over cool-acclimated fungi (Six et al. 2011; Addison et al. 2015; Moore and Six 2015). Warmer temperatures may also alter the ecological landscape of insect hosts indirectly through the volatile compounds produced by warm-tolerant fungal partners that attract parasitoid wasps (Adams and Six 2008; Boone et al. 2008; Six et al. 2011; Addison et al. 2015; Moore and Six 2015). As temperature plays a critical role in mound-building activities of African *Macrotermes* termites and the species of *Termitomyces* fungi with which they associate (Rouland-Lefèvre and Bignell 2002; Vesala et al. 2019), global warming may shift the spectrum of available fungal partners.

- *Agriculture and intentional plant-microbe inoculations*.

Crop plants are dependent on the availability of bacterial and fungal symbionts acquired from the soil in which they are planted. In agriculture, tillage regimes can significantly change the symbiont community composition, reducing the ability of crop plants to find symbiotic partners such as arbuscular mycorrhizal fungi (see e.g., Jansa et al. 2003; Kabir 2005). Conversely and often in response, these soils are often amended with specific biotic (microbial inoculants) or abiotic additions that can shift the availability of strains that can partner with plants more or less efficiently, or with different degrees of long-term stability (Vázquez et al. 2000; Bender et al. 2016; Liu et al. 2020).

(IV) **AC impacts the fitness or ecological interactions of the symbiotic entity as a whole (HOLOBIONT FITNESS)**

This mode focuses on the symbiotic entity as a whole, and how AC impacts the fitness of the holobiont specifically. AC may alter: (i) the niche space or (ii) ecological interactions available to the holobiont as a selectable unit.

(i) *Lichens on gravestones and beneath stained-glass windows—new niches*.

We discussed in relation to mode (I) above how the consequences of human activity, in the form of air pollution, can negatively impact the lichen symbiosis. However, there have also been positive effects of human activity on lichen populations: cemetery gravestones and the leaded windows of churches provided new niches that have spurred the range expansion of diverse lichens (Nash 1989; Seaward 1992, 2004; Purvis and Halls 1996). Lichens colonize gravestone substrates and the areas below stained-glass windows as a result of a confluence of abilities as a holobiont to uniquely occupy these niches: they can attach to stoney substrates, grow photosynthetically, tolerate a range of abiotic stress (e.g., desiccation and UV radiation), and tolerate heavy metal exposure (Backer and Fahselr 2008; Expósito et al. 2020). Lichens grow slowly, but given the very poor fitness of many other biota in these niches, there is apparently little competition or selection for faster growth.

(ii) *Holobiont ecology: lichens, biocrusts, and ectomycorrhizal-pine invasions*.

As a holobiont, lichens serve as a unique food source for grazing deer and caribou, particularly during the winter in northern subarctic forests (Arseneault et al. 1997; Kumpula 2001; Joly et al. 2010). Human introduction and release of these animals in land management efforts have had a profound impact on lichen populations (den Herder et al. 2003; Joly et al. 2009; Klein and Shulski 2011). Biological soil crusts are symbiotic aggregates of fungal and cyanobacterial/algal communities that contribute vital ecosystem services in dryland habitats, including erosion control, soil water retention, and soil nutrient amelioration (Maestre et al. 2013; Pietrasiak 2014; Bowker et al. 2018; Rodríguez-Caballero et al. 2018; Rossi 2020). Biocrust holobionts can be severely disrupted by physical disturbance caused by human foot-traffic or vehicle off-roading. Lastly, human agency in dispersal, intentional species introductions, and management efforts have resulted in not just invasive species, but *invasive assemblages* that thrive in non-native environments. This is well exemplified by the co-introduction of the pine tree and symbiotic ectomycorrhizal fungi, which as a holobiont is more fit in exotic pine habitats than in native habitats (Richardson et al. 2000; Secord 2002; Policelli et al. 2019; Hoeksema et al. 2020).

(V) **AC impacts the trajectory, rhythms, or dynamics of symbiosis (HOLOBIONT TRAJECTORY)**

Although not explicit, modes (I-IV) above largely consider the action of AC in the context of a particular instance in time. However, similar to the discussion above (Section 2.1) on the problems with “instantaneous” or average descriptors of symbiosis, it is important to consider the impact of AC on symbiosis from a “whole trajectory” viewpoint. As symbioses are emergent adaptive systems, disruptions at different points along the formation and developmental timeline of a symbiosis may have disproportionate effects on the persistence and evolutionary trajectory of the symbiosis (cf. Hammond et al. 2020). The examples below highlight how AC disruptions of homeostatic mechanisms or of developmental/life-cycle rhythms can alter the trajectory of symbiosis.

- *Homeostasis and dysbiosis in coral reefs*.

Coral reef ecosystems have homeostatic mechanisms that counter shifts in environmental conditions. However, these can be overwhelmed beyond a tipping point in a non-linear fashion due to inherent feedback loops (Ravindran 2016) in response to AC such as elevated ocean temperatures; this leads to coral bleaching (Weis 2008, 2010) and disease susceptibility (Bruno et al. 2007; Merselis et al. 2018). Even if symbiotic collapse does not occur, significant community compositional shifts may lead to dysbiosis (Egan and Gardiner 2016; Apprill 2017) or alternative states with different partner inter-relationships and degrees of dependence that fundamentally alter the future trajectory of the symbiosis (Putnam et al. 2017; Baker et al. 2018; Allgeier et al. 2020). For example, reduced herbivory due to overfishing may be fundamentally responsible for the shift of some coral reef communities from being coral to macroalgal dominated thus impacting the relative composition of coralline algae that play a foundational role in the coral symbiosis (Holbrook et al. 2016). Anthropogenic nutrient enrichment can also profoundly rewire coral-symbiont relationships to increase holobiont susceptibility to other stressors (Allgeier et al. 2020).

- *Timing of perturbation on symbiotic development and persistence*.

Holobiont sensitivity to AC is very unlikely to be constant over the life cycle of a symbiosis: timing matters. For example, if AC causes the disruption of active mechanisms of symbiotic partners to find each early on or in the call-and-response nature of symbiotic formation (cf. Clear and Hom 2019; Chiu and Paszkowski 2020), it may be hard if not impossible for the symbiosis to be established. Symbioses between partners with biological rhythms may also be prone to having their holobiont trajectories altered by AC. Anthropogenic disturbance of natural daily, lunar, or annual life cycles (e.g., due to artificial light pollution at night) may lead to shifts in coral holobiont evolutionary trajectories (Rosenberg et al. 2019; Ayalon et al. 2019; Levy et al. 2020). This may occur by impacting important processes of reproduction and recruitment (Richmond et al. 2018; Ayalon et al. 2021).

## Symbiogenesis as a consequence and cause of anthropogenic change

We have thus far focused largely on the negative consequences of anthropogenic change on long-established symbioses. However, there may also be positive consequences: AC has the potential to bring about rapid symbiogenesis; novel symbioses (broadly considered) in turn have the potential to cause large-scale environmental change.

Studying natural ecosystems in light of how they might be negatively impacted by AC as an “external force” implicitly assumes that human activity can be decoupled from natural systems. In the Anthropocene, however, in which few ecosystems on Earth can be considered to be untouched by human agency (Plumptre et al. 2021), this assumption may no longer be useful. We must pivot to viewing and understanding neobiota—novel community arrangements and human-affected ecosystems—as the norm (Ellis et al. 2010; Williams et al. 2015). Moreover, rather than viewing symbioses simply as entities subject to AC, *we should consider symbioses as potentially powerful agents of ecosystem change*. Understanding symbiosis with this new perspective will require further work. We discuss several illustrative examples that emphasize this alternative viewpoint.

### Can AC bring about symbiogenesis?

We envision 3 ways in which AC could facilitate the birth of new symbioses: (1) through the creation of novel niches, (2) through the introduction of new partners, and (3) through the formation of new relationships and selection regimes. None of these are mutually exclusive and in fact, symbiogenesis may capitalize on a combination of these 3 schemes. In all schemes, serendipitous complementarity of ‘accidental’ partners may lead to new and unexpected unions on timescales that may be equally rapid as those of AC.

#### New niches for occupancy and for new symbioses to be birthed

AC drives many unprecedented environmental changes that are also opportunities for life to adapt and assemble in new ways. As highlighted in Section 4.2.(IV), symbiotic holobionts may have higher fitness in new niches created by intentional human construction or unintentional anthropogenic mixing. Novel niches provide new opportunities for ecological interactions and partnerships, which may be exploited by new partner pairings. Importantly, these partnerships and potential symbioses may emerge quite quickly without extensive co-evolutionary adaptation, a theme emphasized, for example, by the creation of apparently novel fungal-algal symbioses in the laboratory by engineering environmental conditions conducive for these symbioses to be realized (Aanen and Bisseling 2014; Hom and Murray 2014). The exploitation of new, anthropogenically-driven niches is well demonstrated by the rise of invasive species (Seebens et al. 2017, 2020). In a similar way, new symbioses could form (or be strengthened) through “symbiotic invasions” that exploit new niches (cf. Zhao et al. 2013; Lu et al. 2016; Rassati et al. 2019; Hoeksema et al. 2020). Thus, symbioses should be regarded as important ecological entities if not potential keystones of emerging communities in AC-transformed ecosystems (Lanner 1996; Richardson et al. 2000; Secord 2002; Policelli et al. 2019).

AC can lead to a fundamental shift and conversion of one biome type to another (Foley et al. 2005; Hansen et al. 2013; Hill and Southworth 2016; Watson et al. 2016). For example, increased water demands in agriculture together with global climate change are driving increased desertification of land world-wide (Stringer 2008; Reed and Stringer 2016; Huang et al. 2020). This may open up new habitats for range expansion by desert-favoring symbiotic assemblages like biological soil crusts, which may have an opportunity to colonize. Such new anthropogenically-driven drylands may have their own unique niche characteristics, however, that may select for only specific types of biocrusts (Reed et al. 2016; Steven et al. 2017), or entirely new communities as discussed above (see also Section 5.1.3 below).

#### New partners available for symbiogenesis

Anthropogenic mixing (AM) is a key feature of the Anthropocene. Human mediated dispersal and introduction of foreign species to new areas is an expected consequence of the increased volume and variety of modes of transportation today. Janzen’s concept of **ecological fitting** to explain ecological interactions (Janzen 1980, 1985) applies well here to highlight the potential for new symbioses forming through AM. Ecological fitting can be defined as "the process whereby organisms colonize and persist in novel environments, use novel resources or form novel associations with other species as a result of the suites of traits that they carry at the time they encounter the novel condition" (Agosta and Klemens 2008).

Just as new niche construction may be intentional or unintentional, the pairing of new partners and symbiogenesis can be intentional or unintentional through AM. Laboratory experiments have yielded new symbioses (de-Bashan et al. 2016/4; Jeon and Lorch 1967; Kawabata et al. 1995; Jeon 1995; Kubo et al. 2013; Hom and Murray 2014; Du et al. 2019) and the formation of others may be forthcoming as the field of synthetic ecology grows (Dunham 2007; Grosskopf and Soyer 2014; Kazamia et al. 2014; Song et al. 2014; Zomorrodi and Segrè 2016; Friedman et al. 2017; Cavaliere et al. 2017; Lozano et al. 2019; Kehe et al. 2019; Libby et al. 2019; Mickalide and Kuehn 2019; Hosoda et al. 2020). Agricultural practices to inoculate seeds with ‘probiotic’ cocktails of microbes (O’Callaghan 2016; Rocha et al. 2019) could very well result in novel crop-microbe associations that persist in the soil sufficiently long for co-evolution to occur, although this is rarely if ever investigated to our knowledge. Conceivably, such co-evolution could be facilitated by artificial selection under low- to no-till conditions (cf. Coleman-Derr and Tringe 2014), which is a growing practice today advocated for sustainability reasons (Köhl et al. 2014; Cooper et al. 2016). With synthetic biology methods more commonly deployed to harness or re-engineer nature (for example, the cosmopolitan insect endosymbiont bacterium *Wolbachia* in gene-drive efforts (Wedell et al. 2019; Champer et al. 2020; Carballar-Lejarazú et al. 2020)), unexpected partnerships/symbioses may follow despite intentions and efforts for biocontainment (Lee et al. 2018; Gronvall 2019; Asin-Garcia et al. 2020; Devos et al. 2020).

The rise of new zoonotic diseases as a consequence of AC is well documented (Gottdenker et al. 2014; Han et al. 2016; Gibb et al. 2020) and the birth of new infectious diseases will correlate with the creation of new “parasitic” symbioses. The COVID-19 pandemic has reminded us that the transmission of new human pathogens is greatly facilitated by travel/trade/AM, so much so that in a short amount of time, the SARS-CoV2 virus is on the verge of becoming endemic like influenza (Phillips 2021; Torjesen 2021). Should endemism result, it could be argued that a new virus-human symbiosis has been established. Not all of these virus-human symbioses may be “doom-and-gloom” in nature, however; as more viruses are discovered, new mutualistic viral symbioses may be revealed as well (Roossinck 2015; Roossinck and Bazán 2017).

#### New relationships and selection regimes

In addition to new partners, AC can alter conditions that shift relationships and fundamentally change selection regimes. As discussed in Section 2.1, ecological relationships and pay-off structures of symbioses (or proto-symbioses) can change depending on environmental context, making new metabolic interactions and symbiotic complementarity possible. AC may also impose new selection pressures that favor symbiosis (with physical association) and/or a higher degree of interdependency (cf. Hom and Murray 2014; Gillman 2018).

Human farming, discussed in much greater depth below, may be an archetype for how new relationships and selection regimes have been forged through human agency. In the creation of farms and farming practices, new persistent relationships between humans, crop plants, microbes, invertebrates (e.g., worms, pollinators), and livestock animals have been established and carefully groomed by persistent artificial selection. This has led to both an intimate, proximal/physical co-localization of genetic lineages as well as a deeply interdependent and co-evolving web of interactions between these lineages within the farm ecosystem.

### Symbiosis *sensu lato*: human cultivated systems as novel symbioses?

#### On farming

In Section 4.2, we discussed the impact of AC on some classic examples of symbioses. More broadly, we think it is intriguing and fruitful to consider what other human-created or manipulated systems could be thought of as “symbioses” under the definition articulated in Section 2.1. Human farming systems are an obvious candidate and as discussed later in this section, have profound connections to the Anthropocene. As discussed above, intensive agricultural practices, including the use of selective breeding and artificial fertilizers, have diminished the ability of rhizobia to form beneficial symbioses with crop plants (Porter and Sachs 2020). While some might view this as just another example of the negative impacts of AC on an ancient symbiosis, we favor an alternative (if not provocative) perspective that the essence of this crop plant-nitrogen provisioning symbiosis remains *functionally* the same but with a change in partners: humans have replaced rhizobial symbionts in providing crop plants with nitrogen. This perspective poses a bigger question of whether “farming,” when viewed through the lens of persistent relationships that satisfy complementary *functions*, could be considered a type of symbiosis between humans and specific crops/animals. Our discussion below is framed with the term “farming” as referring to both terrestrial (agricultural) and aquatic (aquacultural) systems, although the majority of examples and focus in the literature (and thus references cited) are of the former.

A number of social and sub-social (i.e., proto-social) insects practice forms of agriculture in which fungal gardens are created, tended, and provide food for their colonies or broods (see Mueller et al. 2005 for a review). This includes the well-known leaf cutter and other attine ants, but also species of termites and ambrosia beetles. These are all longstanding and tightly coupled mutualistic co-evolutionary relationships, commonly described as symbioses. Although human and insect farming are of course different systems, we discuss both below in relation to our definitional criteria for symbiosis. *Could human farming, a paragon of sustained AC, be reasonably considered a type of symbiotic association?*

##### Physical association and a shared environment in farming

In both insect and human farming, one partner constructs and maintains elaborate environments suited to the other cultivated species (Schultz et al. 2005). Both abiotic and biotic aspects of the environment are controlled. Insect farmers create and manage specific habitats for their fungal cultivars, such as chambers within ant and termite nests or networks of tunnels within trees in the case of ambrosia beetles (Mueller et al. 2005). These constructs both buffer against variations of the external environment and reduce potential interactions between the cultivated fungus and other predators or wind-borne pathogens. In many cases, cultivars are provided with special high-value substrates, such as the freshly-cut leaves provided by leaf cutter ants (Benckiser 2010; Hölldobler and Wilson 2010). Humans likewise create specific environments to nurture their crops and livestock, clearing land, ploughing, and fertilizing to encourage crop growth. Farming practices have profoundly altered the environment surrounding human societies, the shape and functions of human society, and human biology itself (see below) (Roosevelt 1984; Larsen 1995; Redman 1999; Bellwood 2004; Lambert 2009; Fitzpatrick 2020). Net positive resource outputs from farming have led to the development of complex constructed environments so that farming is believed to be the reason that human settlements formed, resulting in a shift from the nomadic hunter-gatherer lifestyle (Bellwood 2004; Weisdorf 2005; Thompson et al. 2020; Fitzpatrick 2020). Although most humans and specific domesticated lineages are perhaps not often in “prolonged, direct physical contact” (in contrast to, say, endosymbionts) and only a subset of humans today are directly involved in farming, strong physical *collocation* was crucial for the development and evolution of human-domesticate associations in a self-reinforcing manner. This physical collocation made it possible for repeated, long-term interactions within a shared environment and reduced interactions of all partners with other species, allowing their co-evolution as highly involved associations.

Both insect and human crop farmers actively “plant” inocula cultivars and tend to their cultivated species in an ongoing, trans-generational manner. As in human agriculture, insect farmers perform active maintenance and control of community composition, removing “weed” species that would compete with the primary cultivar of interest for nutrients (Batra and Batra 1979). Attine ants perform intensive monitoring of their fungal gardens and remove diseased cultivars with specialized castes to perform distinct tasks (Currie and Stuart 2001); they also manage an intricate web of associations in the ecosystem that include fungal pathogens and associated beneficial bacteria that produce compounds that counter these pathogens (Currie et al. 2003).

##### Co-evolution and a strong degree of dependency in farming

Insect-fungal agricultural systems are exemplars of strong co-evolutionary interactions with long evolutionary histories of over 20 million years (Chapela et al. 1994; Mueller et al. 2005). There have been many behavioral and anatomical adaptations of insect hosts and their life cycles to allow the construction, planting, and tending of gardens (Traniello and Leuthold 2000; Bot et al. 2001; Hart et al. 2002; Currie et al. 2006) as well as the (often) vertical transmission of cultivar spores when new colonies are founded (Haanstad and Norris 1985; Fernández-Marín et al. 2004). In some cases, the cultivars themselves have evolved to provide nutrition more effectively to their farmers, for example by the growth of nutrient-rich nodules or fungal tip swellings (JRJ and Roeper 1972; Leuthold et al. 1989). For many insect farmers, their dependence on their cultivars is obligate, with cultivars providing the sole source of nutrition for larvae and/or adult insects (Sands 1956; Grassé 1959; Francke-Grosmann 1967; Weber 1972; Mueller et al. 2005)**.** Similarly, the degree of dependence of humans on farmed partners is very high (Larsen 1995; Garibaldi et al. 2011; Granada et al. 2016; Thompson et al. 2020).

The co-evolutionary dynamics in human farm systems is notably characterized by humans that dramatically niche construct and impose strong artificial selection on partner crops and livestock. Although not to the same degree, attine ants also impose some forms of artificial selection to ensure productive and disease-resistant fungal cultivars (Mueller et al. 2004). Human farming has led to the creation of myriad new domesticated species, strains, varieties, and breeds (McCouch 2004; Groeneveld et al. 2010; Brown 2010; Teletchea and Fontaine 2014; Valero et al. 2017; Teletchea 2019). Conversely, there is evidence of genetic change in humans as a consequence of farming (Leach 2003); for example, the ability to digest lactose beyond infancy is associated with pastoralism (Ranciaro et al. 2014) and the ability to digest carbohydrates from marine algae is associated with seaweed farming and consumption (Hehemann et al. 2010).

In general, agriculture and farming can be considered a “ratchet” (Lewis and Maslin 2018). As farming sustains a larger human population (Weisdorf 2005; Gowdy and Krall 2014) and once begun, it must be continued to sustain that population, which in turn nearly always continues to increase. The dependency of humans on any particular crop/animal is usually low to moderate (facultative rather than obligate) since one specific crop may generally be substituted for another, notwithstanding many historical examples of famine due to dependence on monocultures, resource limitations in developing communities, or the cultural importance of particular crops. In insect agriculture by contrast, a particular pairwise relationship is obligate for the farmers. Cultivars are grown as monocultures (Katoh et al. 2002; Aanen et al. 2002) and their removal often causes colony death (Sands 1956; Grassé 1959; Francke-Grosmann 1967; Weber 1972; Mueller et al. 2005). Nevertheless, farmed crops and domesticated animals today are generally highly dependent on human partners: many crops are no longer able to reproduce independently due to genetic changes. For example, staples such as maize and bananas require humans to propagate them and other species such as wheat have been selected for reduced seed dispersal, which facilitates harvesting but likely reduces fitness to live alone with respect to their wild ancestors (Lewis and Maslin 2018). In general, increasing “cultivatability” often leads to a commensurate increase in dependence on humans and the need to be managed, further feeding the farming ratchet as the investment for successful outcomes is increased (Lewis and Maslin 2018). Human dietary changes tied to agricultural practices have led to well-documented reciprocal changes in human genes related to appetite control, metabolic efficiency, and feeding behaviors (Luca et al. 2010)**.**

We suggest that by our definition, both human and insect farming systems can be considered to be symbioses. Striking differences exist between the two, however. While insect agriculture originated multiple times between 20-65 Million years ago (Chapela et al. 1994; Farrell et al. 2001; Aanen et al. 2002), the origins of human farming are a mere 10,000 years ago (Weisdorf 2005). While intricate anatomical adaptations and sequences of behavior have evolved biologically in insect farmers, in human society, equally elaborate farming practices and behaviors are largely cultural and culturally transmitted. The number of human-farmed partnerships is also vast.

Human-domesticate associations share an important feature with many other extant symbioses in allowing the exploitation of new resources and the creation of new niches for the partners involved. These associations divert increasing proportions of primary productivity of ecosystems towards human activities, meeting human needs and appetites, and supporting both human and partner populations (Smil 2011; Williams et al. 2015). This ratchets up an even greater demand for farmed products and farming productivity. This often results in the replacement of extant/wild biomes with anthropogenic biomes (“anthromes” (Ellis et al. 2010)), which is niche construction on a grand scale that often if not always leads to a reduction in biodiversity (Martínez-Ramos et al. 2016; Tilman et al. 2017; Geisen et al. 2019; Sage 2020). Importantly, *this self-reinforcing effect on populations and the large-scale habitat changes that accompany farming efforts, are believed to have played a fundamental role in early anthropic transformation of the biosphere and arguably, the start of the Anthropocene* (Gowdy and Krall 2013, 2014; Lyons et al. 2016; Boivin et al. 2016). As of 2010, approximately 50% of all habitable land has been allocated for agriculture (Ellis et al. 2010; Ritchie and Roser 2013; Williams et al. 2015), a figure that is likely to increase as human population increases.

#### On fermented foods

The practice of food fermentation is believed to predate the origins of agriculture (Steinkraus 2004; Sibbesson 2019; Gänzle 2020) although with increased food yields available through farming, fermentation became a traditional method for preserving surplus crops and animal products during hot summer months when food would quickly spoil, and for consumption in winter months when farm yields were low (Campbell-Platt 1987). Like farming, fermentation—or rather the interaction of humans and microbial fermentation communities—shares some properties of symbiosis in that elaborate processes of environment creation and manipulation by one partner are used to instantiate and maintain microbial partners and ecological processes to their advantage (Steinkraus 2004; Marshall and Mejía-Lorío 2011; Wiest and Schindler 2011; Wolfe and Dutton 2015; Cosetta and Wolfe 2019). These practices became beneficial to human partners in allowing them to leverage the metabolic abilities of microbes to transform (potential) food sources into products that were easier to preserve and transport, circumvented the energetic demands of cooking, increased the bioavailability of nutrients, reduced toxicity, and were reliably and consistently safe to consume (Marshall and Mejía-Lorío 2011; Chaves-López et al. 2014; Gänzle 2020). Below, we explore whether fermentation-based microbial communities can be considered to be in symbiosis with humans in an evolutionary sense, in reference to our definition of symbiosis articulated in Section 2.1. In particular, we address the criteria of: (i) a shared/co-localized and co-constructed environment, (ii) the transmission of lineages (evolutionary persistence), and (iii) reciprocal co-evolution in the lineages.

##### Shared environment and transmission of lineages in fermented foods

It has been suggested that the practice of “intentional” fermentation is very ancient, arising approximately 5 million years ago (and thus vastly pre-dating agriculture) and that it may have played an important role in human evolution (Wiest and Schindler 2011). There is evidence to suggest that plant underground storage organs (i.e., “root vegetables”), such as tubers, were added to the early human (Australopithecine) diet (Wrangham 2009). These are useful sources of carbohydrates and other nutrients, but are often toxic unless processed. As fire was not yet in use and there is little archeological evidence of mechanical processing, intentional fermentation is believed to have been used to detoxify these food sources (Wiest and Schindler 2011).

Such early fermentation practices likely exploited surrounding microbial flora and environments in a spontaneous manner rather than the use of propagated inocula or carefully crafted environments to promote fermentation (Steinkraus 2004; Chaves-López et al. 2014; Tamang et al. 2020). The Huron people processed maize by placing ears of corn into stagnant pools with reducing environments (such as marshes) for several months before cooking and consumption (Tooker 1991; Wiest and Schindler 2011) and is an example of the type of simple process that might have been used to render them edible. The strategy of fermenting food for long periods of time in an effort to detoxify it still persists (Steinkraus 1994; Wiest and Schindler 2011), including that of burying Greenland shark (*Somniosus microcephalus*) meat to produce Icelandic hákarl (Osimani et al. 2019) or fermenting toxic pufferfish ovaries in salted rice-bran paste (Kuda 2015). In this way, fermentation is a means to open up new resources for human sustenance, which has potentially evolutionary consequences.

Fermentation practices and technology have significantly developed and diversified since Neolithic times, including in ways that brought humans into closer association and more extensive interaction with the microbes they now cultivate and propagate. Many culturally codified practices evolved to reliably produce fermented products by constructing effective fermentative niches and developing processes to reproducibly “steer” fermentation towards a desired outcome (Campbell-Platt 1987; Steinkraus 2004; Marshall and Mejía-Lorío 2011). For example, in traditional production of Japanese Koji and derivatives such as sake and doburoku, songs and rhythmic dance movements are used to ensure or precise process timing and very particular physico-sensory cues are used to ensure correct fermentation temperatures or the “doneness” of rice (such as whether cooked rice grains are crushable between finger tips or against a knuckle) (Gekkeikan 1984; Tomoyuki 2018).

As in farming, the practice of fermentation involves the active construction and maintenance of environments (e.g., warmth, darkness) by humans to favor the growth and processes of their desired partners, in this case, beneficial microbial communities. These microbial communities create a characteristically low pH niche that is often facilitated/co-created by humans through the addition of salt to the starting ferment. As in agriculture, fermentation winnows the biodiversity present to those tolerant of the cultivated conditions (in this case, excluding pathogenic microbes). Depending on the complexity of the fermented product (e.g., cheese, wine, Tsukemono-Nukazuke pickles, Chinese Baijiu liquor), there may be several steering steps to ensure a properly controlled and co-created environment for a successful ferment (Hui et al. 2004; Kitamura et al. 2016; Jin et al. 2017; Hutkins 2018). The human drive for fermented products has also led to the construction of physical infrastructures and niches that support their production (e.g., breweries, custom-built cheese caves), arguably in response to the reproducible benefits humans receive from their microbial partners. These built environments may themselves be reservoirs for microbial inocula relevant to the production of the fermented product. Once consumed, the association between microbes resident in the fermented product becomes much more clearly biologically relevant and intimate with human lineages via their gut microbiome. Although vertical transmission of gut microbiota in humans is gaining support (Ferretti et al. 2018; Li et al. 2020), it is unclear at this time if and how fermented products may alter the microbiomes of humans (see below). Nevertheless, microbial cultures handed-down generationally as heirlooms may have led to an entanglement of human and microbial lineages (Ogura 2017; Cook 2018; Flachs and Orkin 2019).

##### Co-evolution and a strong degree of dependency in the production of fermented foods

Culturally, there has been a strong degree of dependency and integration of microbially fermented foods with human society (Wiest and Schindler 2011; Flachs and Orkin 2019); this is revealed by the diversity and amount of fermented food consumption worldwide, with fermented products accounting for nearly a third of contemporary human diets (Campbell-Platt 1994; Dominy 2015). Most fermentation practices developed and evolved over time to become an ongoing human-microbe association (Cook 2018; Flachs and Orkin 2019). However, has this resulted in co-evolved lineages of fermented food microbiota and humans with identifiable genetic change?

Two general categories of fermentation process exist. In spontaneous, “wild” ferments, the fermentation substrate is left open to colonization by local microbiota from raw materials, the surrounding environment, and/or the humans involved in the process. In deliberate, “inoculated” ferments, a fresh fermentation substrate is seeded with a starter culture from a prior batch, a process sometimes referred to as “back-slopping.” Back-slopping for many fermented foods has led to the domestication of microbes with clear genetic signatures of artificial selection, particularly those used in industrial scale fermentations (as demonstrated best in the brewer’s yeast) (Gibbons and Rinker 2015; Gallone et al. 2016; Steensels et al. 2019). Processes using spontaneous fermentation (mostly non-Western ferments (Tamang et al. 2020)) are often extremely elaborate with clearly evolved cultural processes (e.g., Koji ferments) that reliably provide a specific sequence of selection pressures and carefully controlled ecological successions via the provisioning of different substrates/abiotic conditions over the course of fermentation. Such processes, when carried out repeatedly in the same local environment, give rise to genetically distinct microbial communities that can vary even on small spatial scales such as between villages or even producers (Bokulich et al. 2012; Colehour et al. 2014). Similar to heirloom seeds, many microbial starter cultures selected through many human generations of passage are highly prized, jealously (if not secretly) guarded, and usually have high cultural and commercial value.

As “domesticated” microbiomes, it has been proposed that the microbes associated with traditional fermented foods can be considered as part of an “extended genotype” of humans (Bruessow and Brüssow 2020). However, taking a less human-centric view and given our definition above (Section 2.1), could fermented microbial cultures, like domesticated agricultural organisms, be considered to form a symbiosis with humans? We argue yes, especially for (mostly Western) ferments that use perpetually propagated starter cultures such as beer, yogurt, and cheese (Tamang et al. 2020). Even products generated from spontaneous fermentations carried out repeatedly in a site-specific manner over long periods of time show signs of distinct evolutionary genetic changes in the associated microbes (Gibbons and Rinker 2015). Ferments that involve contributions from the human skin microbiome (e.g., sourdough bread (Reese et al. 2020)) or oral microbiome (e.g., Latin American chicha beer (Freire et al. 2016)) highlight an even more intimate association with human fermenters.

But is reciprocal co-evolution occurring for humans in response to their association with these microbial ferments? The processing and preservation of raw foods through fermentation is an ancient form of biotechnology and may have modified human evolutionary pressures by promoting a more diverse diet and by buffering volatility in food provisioning and safety of over time and seasonal changes (Katz 2011; Wollstonecroft 2011). By increasing the bioavailability of nutrients from food sources (Gänzle 2020), fermentation may have been instrumental in facilitating the evolution of larger hominid brains at the expense of smaller guts (Cook 1994; Wollstonecroft 2011; Bryant et al. 2020). A recent study by Peters et al. (2019) suggests that a unique type of hydroxycarboxylic acid receptor (HCA3) associated with improved immune, glucose, and insulin functions may have evolved in humans in response to metabolites specifically produced by lactic acid bacteria (e.g., D-phenyllactic acid) very commonly found in fermented foods (Peters et al. 2019). Fermented foods appear to shape our guts at least transiently (Veiga et al. 2014; Derrien and van Hylckama Vlieg 2015; Taylor et al. 2020) although further studies are needed to determine if such changes in host gut microbiota can become persistent with a steady diet of fermented foods (Stiemsma et al. 2020). We know that geographically distinct diets appear to correlate with compositionally distinct human gut microbiome profiles (e.g., see Conteh and Huang 2020), and that Western and Eastern fermented food microbiota are compositionally distinct (Tamang et al. 2020); however, we currently have few data that clearly link causative, coevolved traits of the host or host microbiome with those of human-passaged microbial ferments. One class of fermented products in particular—alcoholic beverages—has led to a very strong dependency, maintenance, and refinement of microbial cultures that produce these beverages. Although alcoholism is a complex behavioral trait, there is evidence for some evolutionary adaptive changes in certain human lineages in relation to alcohol consumption (Polimanti and Gelernter 2018; Kranzler et al. 2019).

## Outlook

### A short recap

We have chosen to operationally define symbiosis as a physical association between two or more organisms that is distinguished by the following 5 key traits:

1. Symbiotic partners share genetic fates and co-evolve, typically constrained by repeated co-localization if not physical association
2. Symbiotic partners share a uniquely co-created environment for a significant portion of at least one partner’s life cycle
3. There is both a degree of dependency and of functional integration between symbiotic partners
4. A holobiont phenotype emerges from the union of symbiotic partners that is distinct and more than the sum of each partner alone
5. Homeostasis is an emergent property of the union of symbiotic partners that tends to maintain the symbiotic association, and is subsequently a trait for selection.

Rapid changes in both biotic and abiotic conditions at both local or global scales have potential impacts on the ecology and evolution of all species including symbiotic associations. We presented a preliminary typology of anthropogenic change (AC) based on a generalization of known accelerating variables that define the Anthropocene. Our typology of AC (Section 3.2) and framework of AC impacts on symbiosis (Section 4.1) are an initial attempt to unpick and systematically operationalize how different types of environmental change might alter different aspects of symbiotic association, with the ultimate aim of helping us to evaluate how AC could affect different ecological and evolutionary processes relevant to symbioses.

The framework we presented attempts to organize and focus on how specific modes of AC impact on symbioses with respect to individual partner fitness and biotic interactions, the holobiont as an emergent entity subject to unique selective pressures, as well as the symbiotic interaction and the uniquely co-created environment that is characteristic of the symbiotic entity. In particular, we emphasized the need to view symbioses with evolutionary principles firmly in mind, and to embrace a “whole-trajectory” perspective, since symbioses can be ‘fluid’ in both their nature and identity as they bridge the divide between partners-as-individuals and partners-in-union as a new unit of selection. The fluidity of these symbiotic interactions is evidenced by their relational sensitivity to the environment (with respect to the mutualism-parasitism spectrum and the degree of mutual dependency). And yet, symbioses are also often characterized by an emergent homeostasis as partners evolve greater integration (discussed further below in Section 6.2). Together, these features imply that symbioses may be categorically different in their response to AC than other organisms or ecological interactions.

We discussed how AC could be a force for the destruction of extant symbioses (e.g., nitrogen fertilizers negatively impacting rhizobial-legume symbioses) or their expansion (e.g., ectomycorrhizal-pine invasions), as well as for the creation of new symbioses (e.g., new zoonotic virus associations with humans). Symbioses can be profoundly affected by ACs that lead to dramatic changes in selective pressures and the availability of potential partners; symbioses could, however, be resilient to AC and new symbioses may come into being as a result of globally changing trends. We provided select case examples (very far from exhaustive) in Section 4.2 on shifts in symbioses in response to AC and musings on the potential for AC-driven symbiogenesis in Section 5, extending our notion of symbiosis to long-term human endeavors like farming. What we have proposed and discussed is a starting point and meant to prompt future discussion. We believe there are at least 3 key questions to be addressed in understanding the impacts of AC on symbioses that can be drawn out by our conceptualization. Given that symbioses comprise complex sets of biotic-abiotic interactions and the mechanisms to maintain them:

1. *What specific types of AC (Section* *3.2**) are more or less likely to impact symbioses and their evolution?*
2. *What particular aspects of symbioses (Fig.* *2**; Section* *4.1**) are more or less likely to be impacted by a particular type of AC?*
3. *Are symbioses more robust/fragile to AC than other types of ecological relationships or individual organisms? If so, why?*

Tackling these questions will require a large community effort and the study of many different symbiotic systems to provide enough data to enable generalizations that synthesize insights from these systems as a whole. In Section 6.2, we offer several reflections on the last question above on the robustness of symbiosis to AC. We conclude in Section 6.3 with comments on the centrality of symbioses as ecosystem keystones and a call to action for responsible human engagement in the Anthropocene.

### On robustness and function

#### Overview

Implicit in any question about the robustness/fragility of symbiosis to AC is a requisite accounting of the benefits and tradeoffs of forming a symbiosis (i) *relative* to being apo- or non-symbiotic and (ii) *with respect to before and after AC*. The fact that symbioses form and persist implies that they must have been sufficiently fit and beneficial (at least to one partner) to have been selected for under a set of “native” conditions, an idea generally consistent with findings from the limited studies on ancestral reconstructions of symbiotic mutualisms (Sachs et al. 2011; Werner et al. 2014; Maherali et al. 2016; Rimington et al. 2019). The difference between these native conditions and new conditions brought about by AC is a key issue to consider with regards to system robustness. To our knowledge, whether symbioses respond differently or uniquely to AC relative to non-symbiotic ecological interactions remains essentially unexplored in the literature. Before further consideration, however, we review what we know about biological systems, principles of robustness, and the importance of function or processes underlying symbioses.

There are multiple terms—e.g., “robustness,” “resilience,” “adaptive capacity”—that are used differently across various disciplines to describe the capacity for a system to continue to function or exist in response to perturbation or external change. These concepts overlap to some degree and it may be unhelpful to insist on using one or the other. It is critical, however, to articulate *what aspects of the system are to be maintained* (i.e., the robustness of what?). In some cases, this may be so obvious that this issue is not explicitly considered or stated; for example, for an individual organism, “robustness” may simply mean that it remains alive or viable in the face of external change. Regardless, there is a need to specify a timescale and scope, e.g., whether one is concerned with the robustness of the organism over one generation or over many generations with respect to persistence of the evolutionary lineage. More complex biological systems involving multiple individual organisms/units of selection require greater conceptual precision. For example, when we consider whether an ecosystem is robust or resilient to environmental change, we need to specify whether we are concerned with the maintenance of particular ecosystem p*rocesses and functions* or with a specific *community composition*. Symbioses, as mentioned in Section 2.2, embody aspects of an ecosystem and exist along a continuum from flexible but co-evolved ecological relationships to a higher-level evolutionary individual. What we mean in discussions of ***what*** **is changing** (or not) in the face of AC and ***how*** **they change** may be different across this continuum and thus needs to be stated explicitly.

We believe that the conservation of specific instantiations of symbioses (with specific extant partners) may not be as important as the conservation of the ecological functions, processes, and services of those symbioses. Thus, we concur with Doolittle and Booth’s emphasis on the importance of “the song, not the singer”—that it may be more fruitful to cast the “metabolic and developmental interaction *patterns*” (the song) rather than specific taxa (the singers) of a biological collective as the unit of selection that recapitulates such patterns in subsequent generations under the right conditions (Doolittle and Booth 2017). The proposed typology (Section 3.2) and framework (Section 4) should help in generalizing insights from many different symbiotic case studies, and in focusing conceptually on symbiotic patterns; it can free us from being too concerned with the peculiar details of taxonomy or partner identity in specific symbioses, and shift attention to arguably more important details of **process and function**. Our discussion of Fig. 2 outlined the need to think of the persistence of symbioses against AC in light of a set of 5 interconnected targets that must be maintained, any of which could be disrupted.

Biological organisms are “complex systems” in the general sense of being made up of extremely heterogeneous components that are organized into a highly structured network with hierarchies and processes that operate at multiple spatial and temporal scales (Carlson and Doyle 2002). Owing to having evolved this sort of complexity, **living organisms exhibit a property of “robust-*and*-fragile”**: they are robust against some perturbations and fragile to other perturbations, even if small, depending on the structure and feedbacks present within the system (Carlson and Doyle 2002; Kitano and Oda 2006). Feedbacks that develop in complex adaptive systems can be used to maintain system homeostasis in response to common or anticipated perturbations. However, such feedbacks may potentially result in the amplification of small perturbations (often rare or unanticipated) for which the system may not be structured to buffer, which is an unavoidable trade-off (Carlson and Doyle 2002). Thus, *fragility is an intrinsic property for any system composed of biological organisms* and the challenge is how to avoid conditions that resonate with such vulnerability.

Broadly speaking, **“robustness” refers to the ability of a system to maintain essential functions in the face of external perturbation or change** and “resilience” to the capacity of a system to adapt and re-organize in the face of change or perturbation; resilience may also refer to the ability of a system to remain in a given “basin of attraction” of a particular equilibrium state (Resilience Alliance 2010; Capano and Woo 2017; Penn and Barbrook-Johnson 2021). However, robustness and resilience are often used interchangeably, and for our purposes, we are primarily interested in *whether a symbiosis can persist by maintaining key patterns of function, process, and dynamics associated with the holobiont in response to AC*. We describe below how this could occur through redundancy, substitutability, or complementarity of some key functions or components (including partners), active buffering against change (homeostasis), and evolution.

#### Robustness through functional and complementary redundancy

One potential source of robustness comes from having system redundancy or complimentary back-ups of key system components, processes, or functions that provide sufficient internal flexibility or degrees of freedom to respond to a change. This relates in part to Ashby’s “law of requisite variety” from cybernetics and the idea that a stable system must have a sufficient number of states to match the number of states that a problem can present (Ashby 1956). Robust systems can essentially ‘cover’ their weak points by having more components that could compensate should one of those components fail to function. Symbioses are amalgamations of dis-similar organisms, joined together by complementarity but also implicit redundancy. The phenomenon of genome reduction in endosymbionts is a consequence of *functional redundancy* and the notion that duplicative functions will generally be streamlined/trimmed if they do not provide an evolutionary advantage (Delmotte et al. 2006; Moran 2007; Mendonça et al. 2011; Oakeson et al. 2014; Bennett and Moran 2015; Lo et al. 2016). By combining two or more genomes (and depending on the degree of partner dependence and integration), symbioses may be intrinsically redundant in mutually core biological functions that could endow robustness against perturbations that impinge on those functions.

#### Robustness through emergent homeostasis

Symbiotic unions bring not only complementarity and redundancy, but also conflicts and tensions that must be resolved in the coordination of organisms living together and in the negotiation of intrinsic partner self-interests. How these tensions and redundant functions are reconciled with respect to system network, structure, and dynamics to produce homeostasis in the emergent holobiont is precisely the “combinatorial unknown” that makes symbioses a source of evolutionary novelty and open-ended evolution. In addition to the feedback and homeostatic mechanisms that each organismal partner may inherently bring into the symbiosis, new couplings and dynamic responses of partners to one another may instantiate new reciprocal feedbacks in the holobiont system. The co-construction and maintenance of a shared environment is often an important aspect of emergent homeostasis (Fig. 1B) as well as for how we have defined symbiosis (Section 2.1). In addition to helping to ensure genetic co-variance of partner lineages, spatial co-localization and physical association of symbiotic partners can help buffer against external environmental changes by simple proximity and exclusion. Endosymbiosis provides an extreme example where a partner is housed within the body of another (the host) and buffered from the external environment by the internal environment and homeostatic mechanisms of the host. Mechanisms that maintain a shared environment also provide a measure of buffering against the external environment, and hence environmental change (cf. Prada et al. 2017). This is particularly relevant to types 3-5 in our typology of AC (Section 3.2) concerning changes in mean values or the variability of either local or global environmental variables.

Like a buffer system in chemistry, homeostatic buffering capacity operates over an expanded range of external forcing, but will abruptly transition to a new state and potentially fail if pushed beyond a tipping point, a warning consistently voiced by many climate scientists concerning several Earth systems (Lenton et al. 2008; Heinze et al. 2021). How robust this buffering is to AC, i.e., how much change can be absorbed while still maintaining an environment within the bounds that permit continued functioning or persistence of the symbiosis, will depend on a number of factors. These include: (i) the tolerance range of homeostasis, (ii) the timescales of homeostatic regulation, (iii) how responsive buffering mechanisms are to AC, (iv) the fitness costs associated with maintaining homeostasis under conditions of AC, and (v) the duration of AC.

#### Robustness through evolvability

The ability of random genetic variations to sometimes produce fitness improvements is known as “evolvability” (Wagner and Altenberg 1996) and is a property of all living organisms in response to change. It is generally unclear whether the rates of evolution of partners in symbiosis differ substantively relative to those of organisms in other ecological interactions, although this may depend greatly on the nature of the relationship, relative growth rates, strength of selection, and reproductive barriers (cf. Damore and Gore 2011; Brucker and Bordenstein 2012; Bennett and Moran 2015). There is some evidence suggesting, however, that symbiotic associations of bacterial endophytes with coffee plants may have led to increased rates of plant evolution in response to climactic change (Verstraete et al. 2017; Gillman 2018).

As alluded to above with regards to functional redundancy, the joining of dissimilar organisms in symbiogenesis provides an opportunity for rapid evolutionary innovation through new couplings and combinations of functions encoded by partner genomes, of which metabolic functions may be most important (O’Malley 2015). This sudden burst in “functional repertoire” may enable organisms in early stages of symbiogenesis to be more agile or evolvable with respect to AC (assuming coupling benefits outweigh negative tradeoffs). Evolutionarily ‘young’ symbioses may also be subject to more intense and dynamic selection pressures as partners initially adapt to a very different life together in a manner that may facilitate more rapid adaptive changes than in ‘older,’ more well-established symbioses (cf. Delmotte et al. 2006; Oakeson et al. 2014). Thus, the evolutionary age of a symbiosis, as well as the degrees of integration and dependence, may pose key constraints on defining the adaptive capacity of symbioses to evolve in response to selective challenges of AC that impinge on the modes shown in Fig. 2. Unlike homeostasis, which largely deals with transient changes in external conditions, **many facets of AC are about shifting to “new normals”** rather than being brief perturbations. Homeostatic mechanisms may buffer against variations experienced by a symbiosis, but new mean values (e.g., temperature) or greater variation in those values (e.g., hotter and colder temperature extremes and/or greater temporal fluctuations) must be dealt with in a chronic fashion, which likely requires evolutionary adaptation. The tension between homeostasis and evolvability, and the underlying differences in process timescales for both, need to be considered explicitly in models of AC.

As no organism is an island, the landscape of symbiotic evolution will also be strongly influenced by the biotic filter (mode (III), Section 4), which must be factored into considerations of evolvability. For example, the diversity of partners available, the extent to which some partners are “generalists” vs. “specialists” (see Torres-Martínez et al. 2021), and/or whether such interactions are obligate or facultative will have a major impact on the evolutionary trajectory of a symbiosis. Obligate symbioses may be particularly sensitive to AC given that the extinction of one partner would seal the fate of the other(s) (Mayer et al. 2014) unless new surrogate partners are found. For example, as discussed in Section 5.2, over-fertilization is leading to the breakdown of rhizobial-legume crop symbioses, but humans have substituted as the nitrogen provisioning partner for leguminous crop plants (Porter and Sachs 2020). While novel symbioses often lead to range expansion, in general, niche narrowing over time may be expected in obligate, vertically transmitted endosymbionts that can result in fragility with respect to environmental change (Moran 2007; Bennett and Moran 2015).

One might also imagine that obligate mutualisms that are “more open” to environmental variables could be more vulnerable to AC than, for example, those involving endosymbionts in which one partner might shield or buffer the local environment of another through its internal homeostatic mechanisms. A contrasting example is provided by corals: although symbiotic dinoflagellates are endosymbionts of coral tissue, they are unprotected from fluctuating ocean temperatures as the coral host itself generally lacks mechanisms for homeostatic temperature regulation; this can lead to coral bleaching in response to warmer temperatures stemming largely (although not exclusively) from a sensitivity and response of the endosymbionts (Baker et al. 2004; Berkelmans and van Oppen 2006; Sampayo et al. 2008; Weis 2008, 2010; Baird et al. 2009; Cziesielski et al. 2018). For some coral systems, a change in the relative abundance of and dependence on symbiont types within the endosymbiont population in the host can compensate for this temperature fragility (Berkelmans and van Oppen 2006). Coral symbioses are fascinating models for understanding mechanisms for how symbiotic systems could evolve in response to the challenges of AC (Putnam et al. 2017; Stanley and van de Schootbrugge 2018; Blackstone and Golladay 2018; Ying et al. 2018; Weis 2019; Blackstone and Parrin 2020; Buerger et al. 2020; van Oppen and Medina 2020). AC may be inevitable (see below) and efforts that emphasize conserving the *functions and ecosystem services* (“the song”) of coral symbioses may be a more realistic goal than attempting to maintain the specific coral partners (“the singers”) observed today. Thus, projects that aim to understand and support the climate resilience of symbiotic systems through human-assisted evolutionary means (e.g., van Oppen et al. 2015; van Oppen et al. 2017) may be better positioned for success in the Anthropocene than contemporary conservation and management efforts focused merely on preserving existing taxa. Symbiotic associations, functions, and ecosystem services with far-reaching impacts on human well-being may be prone to extinction, not just specific taxonomic lineages (Aslan et al. 2013).

Unfortunately, predicting *a priori* how symbioses could become fragile or sensitive to AC may be incredibly difficult if not impossible. Extant symbioses could provide testable systems for understanding how system redundancy and potential fragility could arise from original, free-living partners, although this would be limited by the degree to which evolutionary and selection histories are known. Regardless, extant symbioses are expected to exhibit robust-and-fragile properties that must simply be studied on a case-by-case basis and analyzed with respect to specific challenges of AC (Section 3.2) before generalization. We believe that a number of fundamental research questions will need to be explored including: (i) under what conditions does symbiotic association (as a life strategy) lead to improved resilience? (ii) how evolvable are different symbioses, and why? and (iii) to what degree are symbiotic associations reversible or canalized and how does this trait impact their evolvability (cf. Bennett and Moran 2015)?

### Why we should care

#### Symbioses as keystones and its broader impacts

From the origin of the eukaryotic cell (Margulis 1992; O’Malley 2015) to the evolution and terrestrialization of land plants (Mills et al. 2018; Delaux and Schornack 2021), symbioses have fundamentally shaped the biodiversity and ecosystems of Earth (Gilbert et al. 2012; Guerrero and Berlanga 2016). Symbioses have also provided critical ecosystem services for humans through structural and metabolic innovations that have become quite wide-spread and dominant, e.g., through coral reefs (Woodhead et al. 2019) and rhizobia-associated leguminous crops and agriculture (de Castro et al. 2016; Stagnari et al. 2017). Given their central role as drivers of ecological and evolutionary change, many symbioses are **keystones of ecosystems** (Secord 2002; Zook 2002) with the capacity to radically transform biomes, open up and occupy new niches (cf. land plants and corals), and fuel ongoing ecosystem expansion through innovative and hypercompetitive resource utilization (cf. invasive fungal-pine symbioses). We discussed how human-crop/livestock farming represents a class of symbioses *sensu lato* that may have jump-started the Anthropocene itself and acts as an expanding ratchet. This self-reinforcing nature of human farming underscores intrinsic autocatalytic properties of all symbioses as ecosystem engineers, no matter the degree or extent. Given positive feedbacks and the number of symbioses tied to biogeochemical cycles that are considered critical for maintaining safe “planetary boundaries” (Rockström et al. 2009), symbioses should be explicitly included in models of global climate change (cf. Wang et al. 2007; Fisher et al. 2010; Nygren et al. 2012; Reed et al. 2015; Aleixo et al. 2020; He et al. 2021).

Although symbiotic associations are clearly important in the local ecosystems in which they are embedded, the impacts of AC on symbioses can be magnified and ripple out to other domains including social systems. We must be mindful of the interrelationships between biological, economic, policy, and social domains in dealing with global climate change and biodiversity loss, which are intimately linked to issues of land use change and intensification, energy production, global inequality, and economic development. Changes within these domains are often compounded by and interact with each other, and social and economic factors on the Earth system may ultimately be more influential than those biological in origin (Donges et al. 2017). Like many key socio-ecological issues, problems arising from AC are so-called “wicked problems” (Rittel and Webber 1973; Levin et al. 2012; Head and Alford 2015), meaning that: (i) effective interventions are extremely hard to determine *a priori*, and (ii) there is no single correct solution to the problem given that there are multiple competing human values. *Wicked problems cannot be “solved,” they can only be navigated and managed, which is the approach we must come to terms with and take to counteract the potential consequences of AC*.

#### The anthropic biosphere is a symbiotic biosphere

The ubiquity and functional impact of symbioses, both traditionally and more broadly considered, suggests a need to consider symbioses more centrally in the future of the anthropic biosphere. We have advocated the perspective that we must frame questions more generally rather than focus merely on specific case examples, no matter how iconic, in order to more effectively advance symbiosis research vis-à-vis the Anthropocene. Human society is intertwined with symbioses on many scales and these relationships have been crucial in our history as a species. For example, as discussed in Section 5.2, human agriculture both depends on plant-microbial symbioses and can itself be considered as consisting of higher-level symbioses with humans and specific domesticated crop species as partners. It is likely that many complex ecological and socio-ecological systems have this “Russian doll” or nested character. We exist in a dynamic, multi-scale landscape in which multiple symbioses with biological, human, and cultural components are interacting, declining, forming and evolving simultaneously. Interdisciplinary approaches for dealing with wicked problems from complexity and social sciences are likely worth considering in research efforts aimed at understanding symbioses in an anthropogenically changing world.

The Anthropocene forces us to re-examine our relationships with the “natural world.” It is not realistic to maintain a perspective that views us as detached from the Earth’s ecosystems, acting merely as custodians or guardians. We have no choice but to “face Gaia” (Latour 2017) and understand that we and the effects of our society are inextricably connected with all ecosystems on Earth today. Less than 3% of our Earth's ecosystems remain untouched by human influence (Williams et al. 2015; Plumptre et al. 2021) and whether we intend to or not, our choices have and will have profound impacts on the future of Earth’s biomes, and through reciprocal feedbacks, our own species. The fact that we have agency (and responsibility) should empower us to take a more active role. There are different paths we could take going forward. Do we focus our interactions with ecosystems towards the preservation of ecosystem services vital to the resilience of human society, the conservation of a “scenic wilderness” that allows our longstanding cultural interactions with ecosystems, to ensure that a particular species will persist, or do we aim to enable extant ecosystems to take on their own evolutionary trajectories into the future wherever that might lead them (Sarrazin and Lecomte 2016)? Will we prioritize ‘the song’ or ‘the singers’? And if the song, which song is it that should be sung?

We believe symbioses are ideal fulcrums for targeted engagement given their keystone nature in ecosystems as described above. Symbiotic associations, even more than keystone species, might be thought of as “ecosystem levers,” able to reconfigure or maintain biomes and abiotic environments and thus numerous niches. We have emphasized a functional view of symbiosis as a network of processes and relationships and presented our view that conservation efforts should maintain the *biodiversity of functions and processes* (“the song”) in light of the realities of AC. Just as important, however, is our co-evolutionary, egalitarian view in which no organism is by definition (or implicit bias) the central controlling agent or primary focus of a symbiotic association. The functions and benefits of a symbiosis are distributed across and emerge from the association as a whole. We must move towards a perspective that fully acknowledges that we ourselves are partners embedded in a symbiotic biosphere within which we must act. Moreover, rather than a heavy-handed approach from “the outside,” in which we use our enhanced understanding of symbioses as levers to “domineer” the ecosystems around us, we advocate more mindful and subtle approaches to “steer and steward” the anthropic biosphere from within in a manner that will require us to understand, respect, and work *with* symbioses. How we develop the understanding and the tools to wisely, sustainably, and humbly do this for the benefit of all on Earth is the great challenge of the Anthropocene before us (cf. Ellis et al. 2021).
